# Improving obstructive sleep apnea screening in patients with atrial fibrillation (SLEEP-AF project)

**DOI:** 10.1016/j.hroo.2026.05.013

**Published:** 2026-05-27

**Authors:** T. Jennifer Walker, Tracy Vernon Platt, Suja Davis, Linda Chappell, Anil Gehi

**Affiliations:** 1 University of North Carolina Health, Chapel Hill, North Carolina; 2 University of North Carolina School of Nursing, Chapel Hill, North Carolina; 3 University of North Carolina School of Medicine, Chapel Hill, North Carolina

**Keywords:** Obstructive sleep apnea, Sleep apnea screening, Sleep study, Atrial fibrillation, Quality improvement

## Abstract

**Background:**

Obstructive sleep apnea (OSA) contributes to negative cardiac remodeling and worsened atrial fibrillation (AF) outcomes, yet screening remains suboptimal despite guideline recommendations.

**Objective:**

This quality improvement project aimed to improve screening, diagnosis, and treatment of OSA in patients with AF.

**Methods:**

A pre- and postintervention design evaluated an OSA screening protocol with home sleep apnea testing (HSAT) and positive airway pressure (PAP) initiation for new patients with AF in the electrophysiology (EP) clinic over 3 months. Patients were screened with an OSA questionnaire; those with elevated scores underwent HSAT coordinated by the EP clinic. Patients with moderate to severe OSA were initiated on PAP therapy.

**Results:**

The sample included 141 patients (pre, n = 81; post, n = 60) with no significant baseline differences. The OSA screening rate increased from 49.3% to 96.7% (*P* < .001), and the sleep study completion rate increased from 2% to 25% over 3 months (*P* < .001). The mean time to diagnosis decreased from 151.5 to 22 days. 15 patients were diagnosed as having OSA; 3 initiated PAP therapy.

**Conclusion:**

This protocol with HSAT and PAP delivered by the EP clinic improved OSA detection and treatment. Further study is needed to understand the impact on patient outcomes.

**Key Findings**

▪ The high prevalence of obstructive sleep apnea (OSA) among patients with atrial fibrillation (AF) underscores the need for improved and systematic OSA screening in this population.
▪ Implementation of a standardized OSA screening protocol within an electrophysiology clinic improved OSA detection and treatment.
▪ Leveraging mailed home sleep apnea tests increased sleep study completion rates (2%–25%) and markedly reduced the time to diagnosis (151.5–22 days).
▪ Allied health professionals play a critical role in leading quality improvement initiatives to optimize outcomes for patients with AF.

## Introduction

Atrial fibrillation (AF) and obstructive sleep apnea (OSA) are both highly prevalent, often coexisting conditions. OSA is a common risk factor for AF, with an estimated prevalence ranging from 21% to 74%.^1^ OSA is characterized by repetitive collapse of the upper airway during sleep. Patients with OSA experience recurrent hypoxia, hypercapnia, and autonomic fluctuations, which can trigger the onset of AF.^1^ Over time, the harmful consequences of OSA on the cardiovascular system, including negative atrial remodeling, elevated right heart pressures, cardiac chamber enlargement, and increased blood pressure, contribute to the development of the cardiac substrate that sustains AF.^1^

Multiple observational studies have shown reduced AF recurrences and lower AF burden in patients with treated sleep apnea than those with untreated or undiagnosed sleep apnea.^1^, ^2^, ^3^ Current AF guidelines recommend screening for and treating OSA as part of comprehensive AF management.^4^ In addition, the American Academy of Sleep Medicine (AASM) recommends that patients with high-risk conditions, including AF, receive routine screening for OSA and emphasizes the importance of diagnosing and treating OSA to improve overall health outcomes.^5^ Despite guideline recommendations, patients with AF are not routinely screened for OSA, and the sleep study completion rate among those referred for sleep apnea testing is often low. This quality improvement (QI) project aimed to improve screening, diagnosis, and treatment of OSA in patients with AF by implementing a standardized OSA screening protocol with coordination of home sleep apnea testing (HSAT) and positive airway pressure (PAP) therapy by the electrophysiology (EP) clinic. The primary aims were to increase the rate of OSA screenings and completed sleep studies by 50% and reduce the time to OSA diagnosis by 50%.

## Methods

### Study design

A pre- and postintervention design was used to evaluate the impact of an OSA screening protocol for new patients with AF in the EP clinic. A retrospective electronic health record (EHR) review was performed to evaluate metrics over a 6-month period before implementation (September 2023 to March 2024) and a 3-month period after implementation (May to August 2024). Pre- and postimplementation data were compared to evaluate process and outcome measures.

### Population and setting

The target population included patients aged 18 years or older with AF presenting to the EP clinic at the University of North Carolina Medical Center. Patients with a previous diagnosis of OSA or sleep-disordered breathing, a previous negative sleep study, or current use of PAP therapy were excluded. The project site is an outpatient EP clinic part of an academic institution located in central North Carolina, comprising 7 EP providers (2 nurse practitioners and 5 physicians) and rotating EP fellows. Additional key personnel include nurses, medical assistants, and an EP nurse coordinator. An average of 23 new patients with AF are seen each month.

### Intervention

#### Screening for OSA

Studies assessing the accuracy of OSA screening questionnaires found that the STOP-Bang questionnaire had the highest sensitivity for predicting OSA (90%; *P* < .05).^6^^,^^7^ The STOP-Bang questionnaire consists of 8 items, with 1 point given for each positive risk factor (snoring, tiredness, observed apnea, high blood pressure, body mass index of >35 kg/m^2^, age >50 years, neck circumference of >40 cm, male gender).^8^ The nursing staff administered the STOP-Bang questionnaire, embedded within the EHR, during the intake process for all new patients seen for AF (Figure 1).

**Figure 1:**
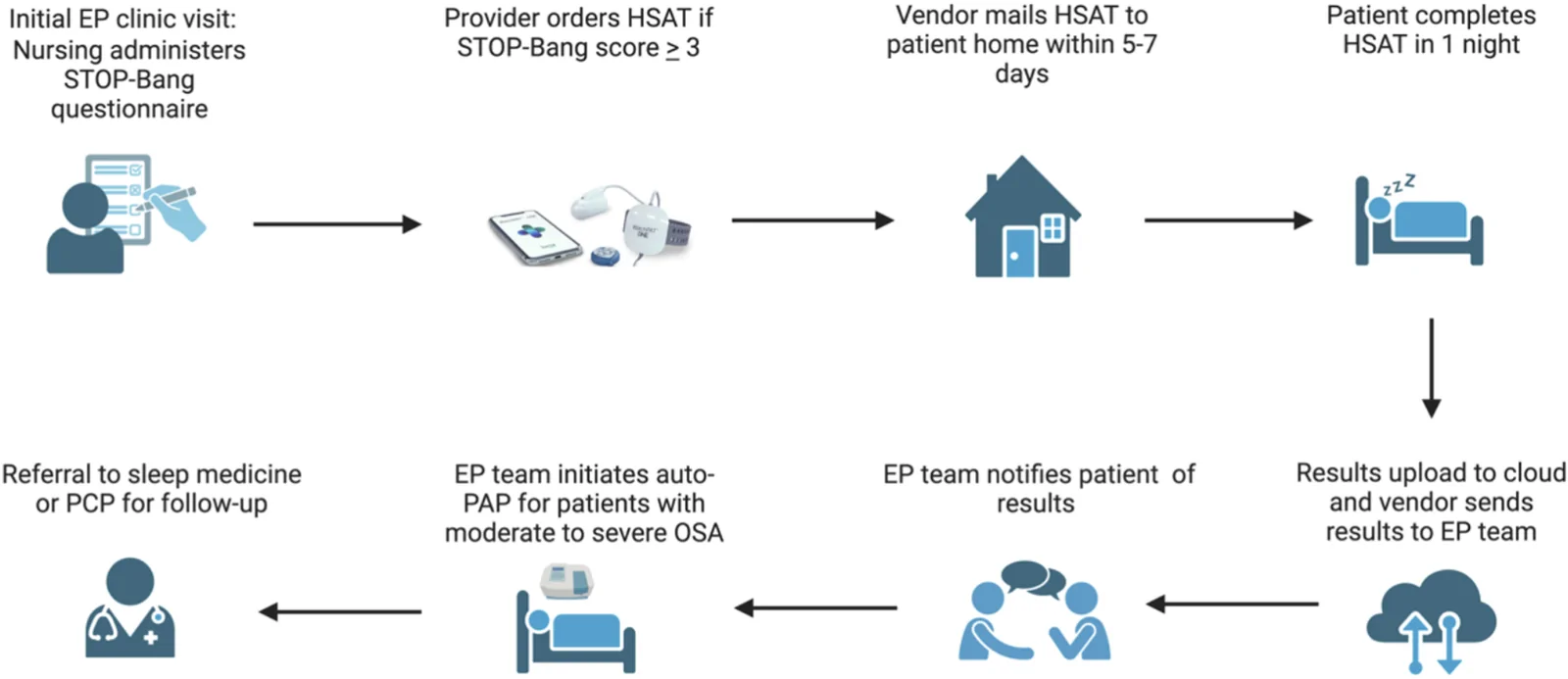
Sleep apnea screening pathway. Created in BioRender. Walker, J. (2026) https://BioRender.com/cewj6s7. Auto-PAP = automatic positive airway pressure; EP = electrophysiology; HSAT = home sleep apnea test; OSA = obstructive sleep apnea.

#### Diagnosis of OSA

For patients at moderate to high risk of OSA (STOP-Bang score ≥3), providers were asked to order a WatchPAT One (Zoll Itamar, Atlanta, GA), which was then mailed to the patient. The WatchPAT One is a disposable home sleep study that uses a chest sensor and peripheral arterial tonometry to detect OSA by measuring physiological pulse volume changes in the finger.^9^ The WatchPAT has been validated in patients with AF, showing a sensitivity of 0.88, specificity of 0.63, and strong correlation with polysomnography-derived apnea-hypopnea index (*r =* 0.80; *P* < .001).^9^

#### Treatment of OSA

The project leader (T.J.W.) reviewed sleep study results and notified patients of results and treatment recommendations through the online patient portal. Patients with mild OSA received electronically delivered education regarding behavioral modifications and were advised to follow up with their primary care provider (PCP). To facilitate treatment for patients with moderate to severe sleep apnea, the project leader placed an order for auto-adjusting PAP (auto-PAP) therapy to a local supplier and coordinated follow-up with either their PCP or sleep medicine. According to the AASM guidelines, continuous PAP and auto-PAP are first-line options for the treatment of OSA, and treatment initiation is not limited to sleep specialists.^10^ The benefits of auto-PAP initiation in the home setting include lower cost, reduced time away from work or home, increased access to care, and faster initiation of treatment.^10^

### Evaluation of the intervention

Baseline data included patients with AF presenting for a new EP clinic visit. Given that no standardized screening protocol was in place, clinic notes and the EHR were manually audited to identify patients who received OSA risk assessment based on clinical suspicion and those who completed a sleep study within 6 months after the initial encounter. A longer follow-up period was allocated to account for historically prolonged sleep study referral and completion timelines.

During the postintervention period, clinic notes and the EHR were reviewed to identify patients who received STOP-Bang screening during the visit and those who completed a sleep study within the study period. Process measures (screenings, provider documentation, referrals, completed HSATs) were tracked and shared with staff and providers weekly to maintain engagement. Outcome measures included the number of patients diagnosed as having OSA, the time from initial consult to finalized sleep study results, and the number of patients who started on auto-PAP therapy.

### Statistical analysis

Pre- and postintervention data were compared. Data analysis was performed using IBM SPSS Statistics software, version 29.0, with a 2-sided *P* < .05 to indicate statistical significance. Continuous variables are presented as mean and standard deviation and were compared using an independent samples *t* test. Categorical variables are presented as percentages and were compared using the χ^2^ or Fisher’s exact test.

### Ethical considerations

The institutional review board for the University of North Carolina at Chapel Hill determined that this QI project did not meet the definition of human subjects research under federal regulations. Patients were given the option to decline the screening, sleep study, or treatment at any time.

## Results

The total sample included 141 patients (pre, n = 81; post, n = 60). There were no significant differences in baseline characteristics between the 2 groups (Table 1). In the preintervention group, the rate of OSA screening by clinical suspicion was 49.3% (n = 40), and 16 patients were referred for further sleep apnea evaluation (1 for a home sleep test, 15 for a sleep medicine consult) (Figure 2). Within 6 months after the initial encounter, 2 patients (2%) completed a sleep study, showing no evidence of sleep apnea. The mean time to diagnosis, from the date of the initial consultation to the finalization of sleep study results, was 151.5 days (range 14–289 days).

**Table 1:** Sample demographics with statistical values

Demographic categories	Preintervention group (n = 81)	Postintervention group (n = 60)	*P* value
Gender			
Female	43 (35)	50 (30)	.424
Male	57 (46)	50 (30)	
Age, y	66.5 ± 15.46	70.8 ± 14	.091
Race			
White	80 (65)	78 (47)	
African American	11 (9)	17 (10)	
Hispanic	2.4 (2)	3.3 (2)	
Other	6.2 (5)	1.7 (1)	
AF type			
Paroxysmal	63 (51)	67 (40)	.649
Nonparoxysmal	37 (30)	33 (20)	
HTN	66.7 (54)	70 (42)	.675
CAD	19 (15)	8 (5)	.087
HFrEF	7.4 (6)	1.66 (1)	.239
Obesity	33.3 (27)	33.3 (20)	1
BMI (kg/m^2^)	28.33 ± 5.73	28.4 ± 7.24	.951

Values are mean ± standard deviation or % (n). Continuous variables (age, BMI) were compared using an independent samples *t* test. Categorical variables were compared using the χ^2^ test (gender, AF type, HTN, CAD, obesity) or Fisher’s exact test (HFrEF).

AF = atrial fibrillation; BMI = body mass index; CAD = coronary artery disease; HFrEF = heart failure with reduced ejection fraction; HTN = hypertension.

**Figure 2:**
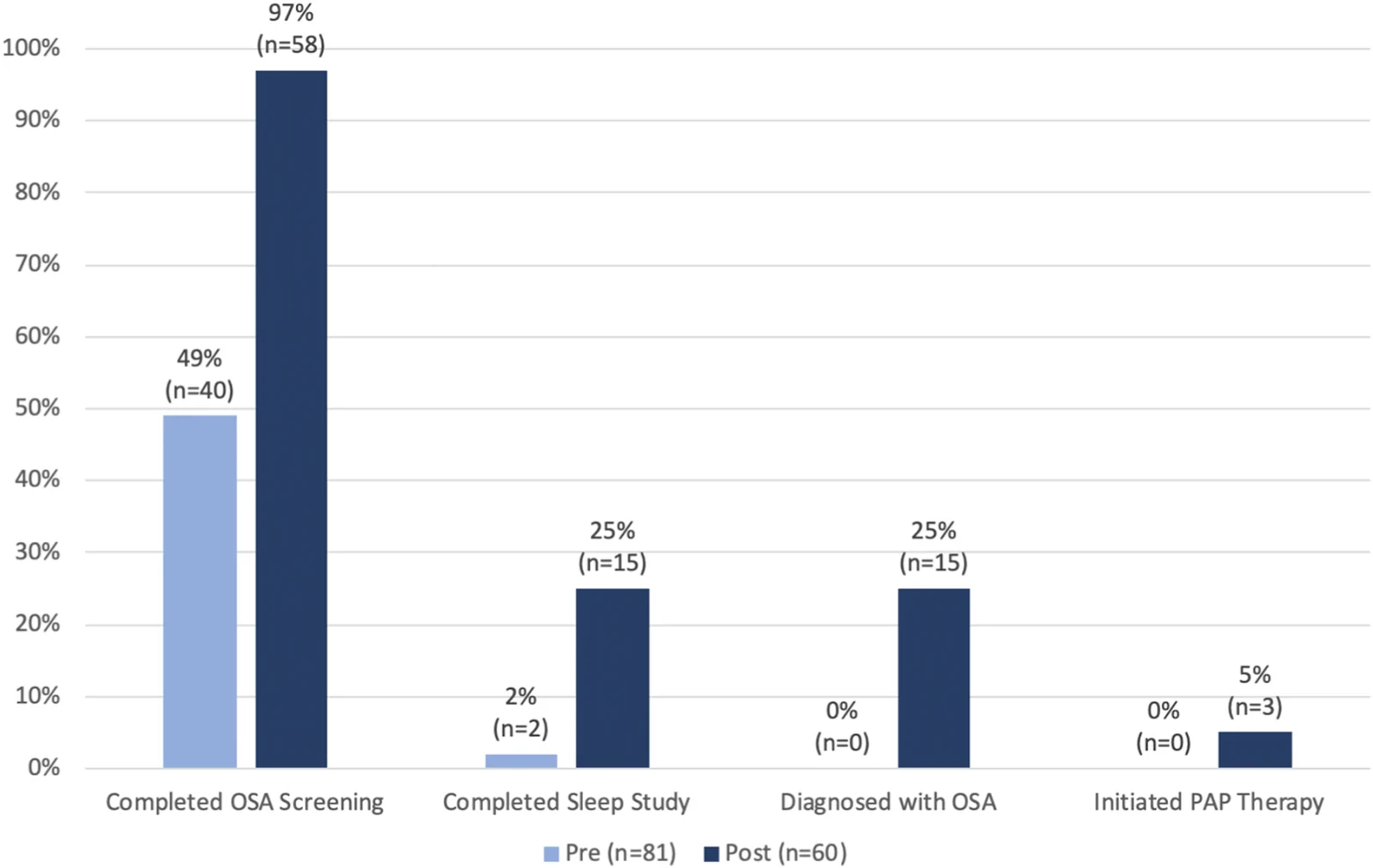
Pre- vs postintervention metrics. The OSA screening rate increased from 49.3% to 96.7% (*P* < .001), and the sleep study completion rate increased from 2% to 25% (*P* < .001). After the intervention, 15 patients were diagnosed as having OSA; 3 initiated PAP therapy. OSA = obstructive sleep apnea; PAP = positive airway pressure.

During the implementation period, screening using the STOP-Bang questionnaire was performed on 96.67% of patients (n = 58). The mean STOP-Bang score was 3.66 ± 1.67, and 75% of patients (n = 45) scored 3 or higher. 27 of the 45 patients (60%) with elevated STOP-Bang scores were referred for further evaluation with a home sleep test (n = 25) or a sleep medicine consult (n = 2). Provider documentation of OSA risk assessment or STOP-Bang screening was completed 87% of the time.

Of the 25 patients referred for HSAT, 15 patients completed the study. Reasons cited for incompletion included insurance denial (n = 1), inability to add the application to the smartphone (n = 1), lack of smartphone (n = 1), and spotty Internet (n = 1). 6 patients did not provide reasons for not completing the home sleep study. All 15 patients who completed the sleep study were diagnosed as having sleep apnea (6 with mild OSA, 6 with moderate OSA, and 3 with severe OSA). The mean time to diagnosis was 22 days (range 9–56 days). Among the 6 diagnosed as having mild OSA, education regarding behavioral modifications (eg, weight loss, avoiding alcohol or sedatives at bedtime, avoiding supine sleep position) was provided, and they were encouraged to follow up with their PCP. Of the 9 patients diagnosed as having moderate to severe OSA, 2 were referred directly to sleep medicine owing to complex features (mixed obstructive and central sleep apnea or financial constraints). The remaining 7 were referred directly for auto-PAP therapy, and follow-up was arranged with their PCP or sleep medicine. 3 patients (5%) were successfully initiated on auto-PAP therapy.

## Discussion

Given that the prevalence of AF in the United States is projected to reach 12 million by 2030,^4^ efforts to improve the management of AF risk factors, including sleep apnea, are essential to improving patient outcomes. This study aligns with the AASM and American College of Cardiology/American Heart Association/American College of Clinical Pharmacy/Heart Rhythm Society AF guideline recommendations to screen for and treat OSA in patients with AF.^4^, ^5^, ^6^^,^^10^ We demonstrate successful implementation of a standardized, allied health-led approach to improving OSA screening and detection, meeting all 3 primary aims (Table 2). The OSA screening rate nearly doubled, from 49.3% before to 96.7% after intervention. Completion of sleep studies increased more than 10-fold from 2% to 25%, and the mean time to diagnosis decreased markedly from 151.5 to 22 days. Of 60 patients in the intervention period, 15 (25%) were diagnosed as having OSA and provided treatment recommendations.

**Table 2:** Primary aimsPrimary aims

	Preintervention group (n = 81)	Postintervention group (n = 60)	% change
1. Increase the percentage of OSA screenings by 50%.	49%	97%	96% increase; *P* < .001
2. Increase the percentage of completed sleep studies by 50%.	2%	25%	1150% increase; *P* < .001
3. Reduce time to diagnosis by 50%.	151.5 d	22 d	85.5% decrease

OSA = obstructive sleep apnea.

Our study has several important implications. The high incidence of OSA in this study emphasizes the importance of routine screening for OSA in patients with AF. Beyond its complex association with AF, OSA is an independent risk factor for cardiovascular disease, including hypertension, heart failure, coronary artery disease, stroke, and increased mortality.^3^ In our sample, the most common comorbidity was hypertension (68%), followed by obesity (33%) and coronary artery disease (14%). Because these comorbidities frequently coexist, early identification and treatment of OSA may provide benefits beyond AF, including mitigation of negative cardiac remodeling and improvements in blood pressure, sleep quality, metabolic regulation, and weight.^2^^,^^6^ These synergistic effects on risk factor modification are a cornerstone of AF management.^4^ OSA treatment may also affect the efficacy of rhythm control strategies, including catheter ablation. Meta-analyses of more than 1000 patients show that untreated OSA is associated with a 31% higher AF recurrence rate after pulmonary vein isolation.^1^ In patients with AF with OSA undergoing pulmonary vein isolation, treatment with PAP therapy is associated with a 42% reduction in AF recurrence.^2^ These potential downstream effects of OSA on AF outcomes highlight the importance of integrating OSA screening into clinical workflows.

Despite the high prevalence of OSA and the importance of diagnosis and treatment, our study demonstrates that OSA is frequently undiagnosed in an AF population when there is no systematic screening process in place. Key barriers to routine OSA screening include the absence of a structured process, time constraints, lack of clinical decision support, insufficient resources, variations in practice patterns, and patient refusal.^11^ Furthermore, limited access to sleep clinics and barriers to polysomnography, such as fear of the overnight stay, cost, and transportation, contribute to the underdiagnosis of OSA.^11^ The AASM recognizes home-based studies, including the WatchPAT, as reasonable options for diagnosing OSA in adults without complicating features (eg, neurologic disease, severe cardiopulmonary disease).^6^ The WatchPAT system requires access to a smartphone application, which was a barrier for 2 patients. Despite this technological requirement, the increase in sleep study completion from 2% to 25% in our study suggests that the mailed, disposable home sleep studies were an effective strategy. Implementing a standardized approach to OSA screening and leveraging mailed home sleep studies may help bridge this significant gap in the care of patients with AF.

Although this workflow was implemented in a single clinical setting, a similar workflow could be adopted across most clinical settings with only minor modifications. Notably, it requires only clinical personnel time without the need for additional equipment. By establishing partnerships with vendors who can provide HSAT and PAP therapy, this care pathway can be implemented for most patients within EP or cardiology practices. The level of engagement with a sleep center may vary, ranging from early involvement to consultation only for complex patients.

### Strengths

Previous studies focused on improving OSA screening and increasing referrals for sleep evaluation in settings such as primary care or general cardiology clinics, as well as using virtual pathways for HSAT.^11^, ^12^, ^13^ At present, this is the first allied health-led QI project to implement OSA screening using a novel approach that includes coordination of HSAT and auto-PAP therapy in patients with AF by an EP clinic. This study highlights the important role of allied health professionals in leading QI initiatives to optimize outcomes for patients with AF.

### Limitations

First, the short project duration, small sample size, predominantly Caucasian sample, and single-site setting may limit the generalizability of the findings. The imbalance in follow-up time in the pre- and postintervention periods was because of time constraints of project support and may have affected the findings. Despite the shorter follow-up time in the postintervention period, there was still a substantial impact seen with the intervention. Second, the WatchPAT device requires an application to be downloaded on a smartphone, which may pose a technological barrier for some patients. Third, 10 of the 25 patients referred for the sleep study did not complete the study, and 4 referred for PAP therapy declined treatment, underscoring persistent challenges with patient acceptance of both diagnosis and treatment. Reasons reported for not starting treatment included skepticism of the results, desire to try behavioral modifications first, and desire to prioritize treatment of other health conditions. Studies examining barriers to acceptance of PAP therapy include additional reasons such as lack of perceived benefit, lack of symptoms, cost, and mask intolerance.^14^ In a future phase of this project, we plan to enhance patient education on the impact of OSA on AF and cardiovascular health to increase patient acceptance of OSA treatment. In addition, we hope to strengthen collaboration with sleep specialists who may provide alternative options such as oral appliance therapy, surgical intervention, or weight-loss pharmacotherapy for patients reluctant to use PAP therapy. Finally, dependency on the project leader could affect the project’s sustainability and scalability. Importantly, integration between the vendor’s platform and the EHR could streamline workflow, reduce workload, and support sustainability.

## Conclusion

The high prevalence of OSA emphasizes the importance of improving OSA screening practices in patients with AF. A novel OSA screening protocol with HSAT and auto-PAP initiation by the EP clinic successfully improved the detection of OSA and reduced the time to OSA diagnosis in patients with AF. Arrhythmia centers should consider implementing a standardized OSA screening protocol as part of comprehensive AF management.

## Disclosures

Dr Gehi reports research funding from the Bristol Myers Squib Foundation. The other authors have no conflicts of interest to disclose.
